# Essential Role of Mast Cells in the Visceral Hyperalgesia Induced by *T. spiralis* Infection and Stress in Rats

**DOI:** 10.1155/2012/294070

**Published:** 2012-03-07

**Authors:** Chang-Qing Yang, Yan-Yu Wei, Chan-Juan Zhong, Li-Ping Duan

**Affiliations:** Department of Gastroenterology, Peking University Third Hospital, 49 North Garden Road, Haidian District, Beijing 100191, China

## Abstract

Mast cells (MCs) deficient rats (Ws/Ws) were used to investigate the roles of MCs in visceral hyperalgesia. Ws/Ws and wild control (+/+) rats were exposed to *T. spiralis* or submitted to acute cold restraint stress (ACRS). Levels of proteinase-activated receptor 2 (PAR2) and nerve growth factor (NGF) were determined by immunoblots and RT-PCR analysis, and the putative signal pathways including phosphorylated extracellular-regulated kinase (pERK1/2) and transient receptor potential vanilloid receptor 1 (TRPV1) were further identified. Visceral hyperalgesia triggered by ACRS was observed only in +/+ rats. The increased expression of PAR2 and NGF was observed only in +/+ rats induced by *T. spiralis* and ACRS. The activation of pERK1/2 induced by ACRS occurred only in +/+ rats. However, a significant increase of TRPV1 induced by *T. spiralis* and ACRS was observed only in +/+ rats. The activation of PAR2 and NGF via both TRPV1 and pERK1/2 signal pathway is dependent on MCs in ACRS-induced visceral hyperalgesia rats.

## 1. Introduction

Irritable bowel syndrome (IBS) is a common gastrointestinal disorder seen by gastroenterologists. Patients classically present with chronic abdominal pain, associated with an alteration in bowel habits, and visceral hyperalgesia which is generally considered to be a hallmark of IBS by their lowered thresholds for pain, increased intensity of sensations, and/or exaggerated visceromotor response to colorectal distension (CRD) [1, 2]; however, the underlying pathogeneses of visceral hyperalgesia are still unknown. 

At present, growing evidences have indicated that mast cells (MCs) play an important role in visceral hyperalgesia [3, 4]. MCs take part of host defense against parasitic and bacterial infections. A subset of patients with IBS have an increased number of MCs in the colonic mucosa [5]. It has also been shown that MCs infiltration and release of mediators in the close proximity of mucosal innervation may contribute to abdominal pain perception in IBS patients [6]. Accumulated evidences have indicated that MCs tryptase is known to be involved in promoting pain and visceral hyperalgesia by activating the proteinase-activated receptor 2 (PAR2) which is expressed on primary afferent nociceptive neurons [7]. Nerve growth factor (NGF) can be released from MCs due to stimulus-induced degranulation, which also plays a pivotal role in colonic hyperalgesia [8].

On the processing of signal transduction of pain sensation, the transient receptor potential vanilloid receptor 1 (TRPV1) is also expressed and colocalized with PAR2 on C-fiber primary sensory afferent neurons [9]. Previous studies have shown that upregulation of PAR2 and NGF enhance the activation of TRPV1 channel [10]. Moreover, as a sensor for thermal and acidic nociception, TRPV1 plays critical roles in the processing of visceral inflammatory pain [11]. It has been demonstrated that pancreatic pronociceptive stimuli with PAR2 agonists cause extracellular-regulated kinase (ERK 1 and ERK2) phosphorylation in the spinal dorsal horn through activation of TRPV1 channels [12]. Noxious stimuli cause phosphorylation of ERK (pERK) in the afferent neuron that contributes to facilitation of pain sensation and is often used as an immediate marker for excitation of afferent neuron following colonic nociception [12]. It has been revealed that NGF activates ERK1/2 and pERK1/2 inhibition decreases excitability in DRG neurons in culture [13]. Although the relationship between visceral hyperalgesia and MCs in IBS animal models and patients has been reported from several laboratories, the essential role of MCs in the progress of various stimulate is not well understood. In the present study, on the basis of using MCs deficient rats, we attempted to identify colonic (PAR2 and NGF) and peripheral sensory neuronic alterations (pERK1/2 and TRPV1) that can be involved in the visceral hyperalgesia triggered by both intestinal infection and stress.

## 2. Materials and Methods

### 2.1. Animals

 Male MCs deficient rats (Ws/Ws) and their normal wild-type littermates (+/+) were obtained from TGC Inc. (Kanagawa, Japan). Rats were housed in standard polypropylene cages containing 2.5 cm of wood chip bedding material, which was maintained at 22°C with an automatic 12 hour light/dark cycle. Rats received a standard laboratory diet and tap water ad libitum. The experiments were conducted when the rats reached approximately 12 weeks of age. All procedures were aimed to minimize both animal number and suffering of the animals and were approved by the Animal Care Committee of Peking University.

### 2.2. *T. spiralis* Induced Colitis

Rats were induced by administering 1.0 mL of 0.9% saline solution containing 1500 *T. spiralis* larvae by gavage. An equivalent volume of vehicle (saline) was administered into control rats. The postinfection (PI) rats were allowed to have a recovery during 100 days period following administration.

### 2.3. Acute Cold Restraint Stress Procedure (ACRS)

 Briefly [14], 100 days after recovery, one half of the control and one half of the PI rats were restrained in individual polymethyl methacrylate restraint cages, and these animals were designated as ACRS and PI  +  ACRS groups. The animals were then placed in their cold home cages at 4°C for 2 hour. ACRS was routinely performed between 10:00 AM and 12:00 AM.

### 2.4. Visceromotor Response to CRD

Sensitivity to CRD was determined using the abdominal withdrawal reflex (AWR) as previously widely described. The rats received a standard CRD procedure, and the first balloon dilation used was 1 mL, and then increasing phases of distension (0.2 mL ascending increments) were applied for 20 second every 5 minutes until the AWR score reached 3. This evaluation was performed by three independent observers, and the AWR score was assigned as follows: 0: no behavioral response to distension; 1: brief head movements followed by cessation of movement; 2: contraction of abdominal muscle without lifting of abdomen; 3: lifting of abdomen; 4: body arching and lifting of pelvic structure.

### 2.5. Immunofluorescence

 L6S1 DRGs segments were removed and fixed for overnight in 4% paraformaldehyde (PFA) in 0.1 mol/L phosphate buffer saline (PBS) at 4°C and then cryoprotected overnight in 30% sucrose in PBS. The tissue was embedded in Tissue-Tek OCT compound medium (Sakura Finetek, USA) and frozen in isopentane at −45°C. Cryostat sections (10 *μ*m) were postfixed with acetone (10 minute, −20°C), and permeabilised with 0.3% Triton X-100 for 2 hours, and then blocked with 10% normal goat serum in PBS with 0.3% Triton X-100 for 30 minutes at room temperature. Sections were incubated overnight at 4°C with rat anti-TRPV1 (1 : 500; Chemicon) and rabbit anti-phospho (p) ERK1/2 (pERK; 1 : 1000; Cell Signaling Technology). Sections were then washed with PBS and incubated for 30 minutes at 37°C with FITC-conjugated goat anti-rat (1 : 100) and FITC-conjugated goat anti-rabbit (1 : 100) antibodies (Sigma). Sections were used to counterstain with DAPI-fluoromount (SouthernBiotech, USA). Digital images of five slices per individual DRG per animal were captured under the same parameters in the fluorescence microscope (Leica DM3000, Leica Microsystems, Germany) at lower magnification (×200 objective). The mean gray level intensity for a region of interest of the images was determined by using Image Pro Plus 6.0 image analysis software system (Media cybernetics, Silver Spring, MD, USA).

### 2.6. Western Blot Analysis

 L6S1 DRGs and distal colon were dissected, and the samples were homogenized in ice-cold RIPA lysis buffer containing 50 mmol/L Tris, pH 7.4, 150 mmol/L NaCl, 1% v/v Triton X-100, 1% sodium deoxycholate, 1% SDS, and “Complete,” mini, EDTA-free protease inhibitor cocktail (Roche Diagnostics, Germany). Proteins were separated in 10% SDS-polyacrylamide gel electrophoresis and then transferred to PVDF membranes (BioRad). Nonspecific binding sites were blocked for 1 hour with 5% nonfat milk in tris-buffered saline Tween-20 (TBST). The blots were then incubated overnight at 4°C with the following primary antibodies in 2% nonfat milk in TBST : PAR2 (1 : 200; Chemicon); rabbit anti-p44/42 MAPK (1 : 1000; Cell Signaling Technology, MA, USA); phospho-p44/42 (Thr202/Tyr204) MAPK (1 : 1000; Cell Signaling Technology, MA, USA); rat anti-TRPV1 (1 : 1,000, Chemicon); and rabbit anti-*β*-actin (1 : 2,000; CWBiotech; China). The membranes were then incubated in appropriate secondary antibodies (IRDye 800CW conjugated goat-anti-rat IgG, 1 : 10,000, or goat-anti-rabbit IgG, 1 : 10,000, li-cor, USA) for 1 hour at room temperature in darkness. Images of the bands in the membranes were captured and analyzed with a Licor odyssey scanner (Licor Biotechnology, USA). The relative expression of each protein was calculated as the ratio of signal density to *β*-actin density. The mean value of the two bands (pERK1 and pERK2) was calculated and normalized with the loading control (total ERK).

### 2.7. RT-PCR

The RNA extraction from colonic tissues was carried out using the RNeasy Micro Kit (QIAGEN) according to manufacturer's instructions. RNA concentration was determined by absorbance at 260 nm, and its integrity was verified by electrophoresis. The first-strand cDNA was synthesized from 2 *μ*L of total RNA by using SuperScript II RNase H reverse transcription (Invitrogen) and oligo- (dT) 12–18 primers according to its protocol. Quantitative PCR was carried out using following primer while using 18S ribosomal RNA as an endogenous control. Samples were amplified in duplicate using the following thermal cycling conditions: 95°C for 3 minutes followed by 40 cycles of amplification at 95°C for 15 seconds and then 60°C for 1 minute to allow for denaturation and annealing-extension. After amplification, a dissociation curve was plotted against melting temperature to ensure amplification of a single product. Comparative cycle threshold values were recorded, and the relative expression of mRNA species was then quantified in duplicate using the 2^−ΔΔCT^ method using iCycler optical system interface software (v2.0, Bio-Rad).

Primers for target genes included the followings:

**Table:** 

NGF	Sense: 5′ AGCGTAATGTCCATGTTGTTCTACA 3′
	Antisense: 5′ TGTCAAGGGAATGCTGAAGTTTAGT 3′
PAR2	Sense: 5′ CCGAACGAAGAAGAAGCACCCT 3′
	Antisense: 5′ GGAGCAGTACATATTGCCGTAGAAA 3′
TRPV1	Sense: 5′ TGGTACTGTACTTCAGCCAACGC 3′
	Antisense: 5′ GAACACGAGGTAGACGAACATAAA 3′
18S rRNA	Sense: 5′ GTAACCCGTTGAACCCCATT 3′
	Antisense: 5′ CCATCCAATCGGTAGTAGCG 3′

### 2.8. Statistical Analysis

Data were presented as mean ± standard error of the mean (SEM). The statistical significance of data was determined using one-way analysis of variance (ANOVA) followed by least significant difference (LSD) or the Student-Newman-Keuls (S-N-K) tests. Statistical calculations were performed using SPSS for windows (version 13.0; SPSS Inc., IL, USA). A *P* value of <0.05 was considered significant in all instances.

## 3. Results

### 3.1. Visceral Hyperalgesia Induced by ACRS Dependent on MCs

**Figure 1:**
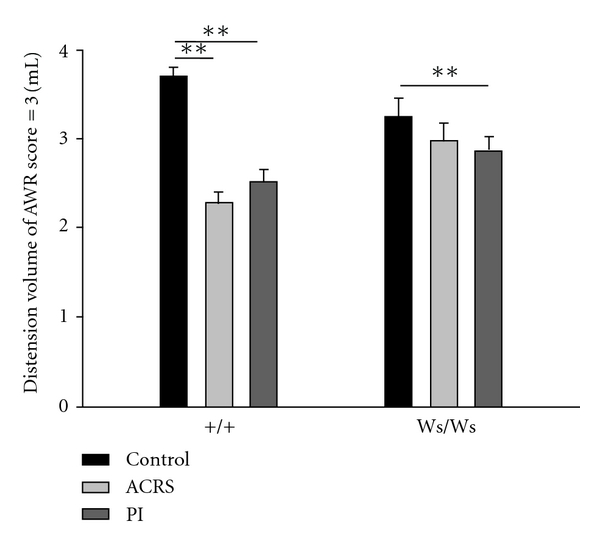
The distension volumes needed to reach an abdominal withdrawal reflex (AWR) score of 3 were significantly lower in postinfection (PI) rats, rats induced by acute cold restraint stress (ACRS), and PI rats received ACRS procedures (PI + ACRS). Each group represents the mean ± SEM of 6 rats. **P < 0.01.

In response to CRD, the distension volume to reach AWR score = 3 was significantly lower in +/+ rats triggered by transient *T. spiralis* intestinal infection (*P* < 0.01) and ACRS (*P* < 0.01). Although *T. spiralis* intestinal infection decreases visceral threshold of pain sensitivity to CRD in Ws/Ws rats (*P* < 0.01), it seems to be inoperative for Ws/Ws rats triggered by ACRS (Figure 1).

### 3.2. The Increased Transcription and Expression of PAR2 Induced by *T. spiralis* Infection and ACRS in Distal Colon Dependent on MCs

**Figure 2:**
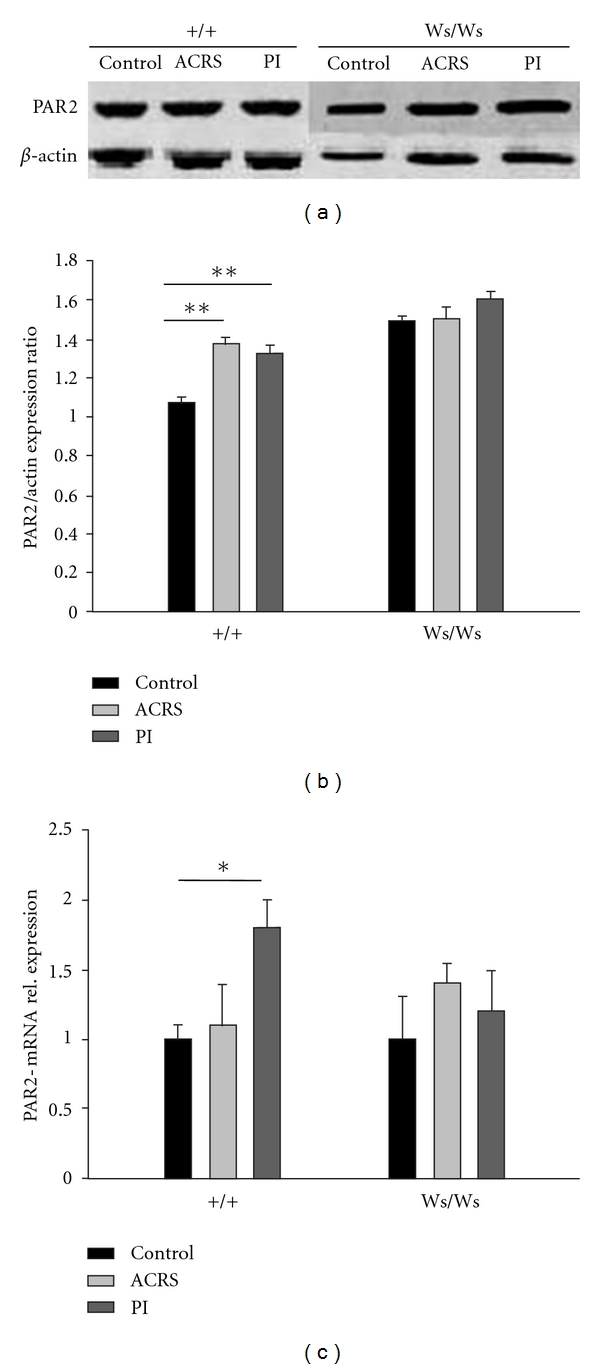
The increased PAR2 expression induced by intestinal infection (PI) and acute cold restraint stress (ACRS) in distal colon of +/+, but not Ws/Ws, rats. (a) Representative western blotting for PAR2 in extracts from colon tissue. (b) Quantitative analysis of PAR2 protein. Data was expressed as normalized density to *β*-actin. (c) Relative levels of PAR2 mRNA in colon tissue. Data was normalized to 18S ribosomal RNA and expressed using the 2^−ΔΔCt^ method. *P < 0.05; **P < 0.01.

The changes in PAR2 expression in rats were confirmed using western blot analysis. As shown in Figures 2(a) and 2(b), the protein level of PAR2 increased to 1.38 ± 0.03 (*P* = 0.001) and 1.32 ± 0.04 (*P* = 0.004) in distal colon in +/+ rats induced by ACRS, and *T. spiralis* intestinal infection, respectively, compares with controls (1.07 ± 0.03) by quantitative densitometry of the immunoblots. Although we only see a significant difference (*P* = 0.036) between the PI group (1.82 ± 0.15) and control group (1.00 ± 0.09) in +/+ rats (Figure 2(c)) in PAR2 mRNA levels, neither ACRS nor *T. spiralis* intestinal infection had an effect on both PAR2 protein and mRNA levels in Ws/Ws rat.

### 3.3. The Increased NGF mRNA Levels Induced by *T. spiralis* Infection and ACRS in Distal Colon Dependent on MCs

**Figure 3:**
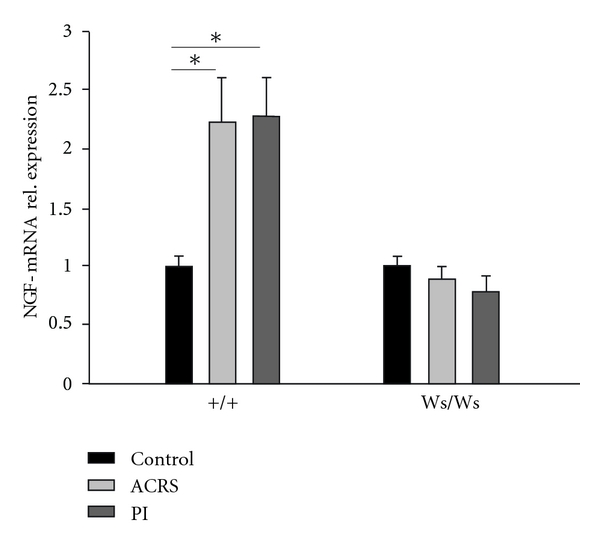
Relative levels of NGF mRNA were increased in the distal colon in +/+ but not Ws/Ws rats induced by intestinal infection (PI) and acute cold restraint stress (ACRS). Data was normalized to 18S ribosomal RNA and expressed using the 2^−ΔΔCt^ method. *P < 0.05.

Compared with control (1.000 ± 0.079), a significant upregulation of NGF mRNA induced by transient *T. spiralis* intestinal infection (2.274 ± 0.338, *P* = 0.010) and ACRS (2.213 ± 0.397, *P* = 0.014) in the distal colon in +/+ rats. However, the NGF levels induced by transient *T. spiralis* intestinal infection and ACRS have not been changed in Ws/Ws rats (Figure 3). 

### 3.4. ACRS Evokes Phosphorylation of ERK in L6S1 DRGs Dependent on MCs

**Figure 4:**
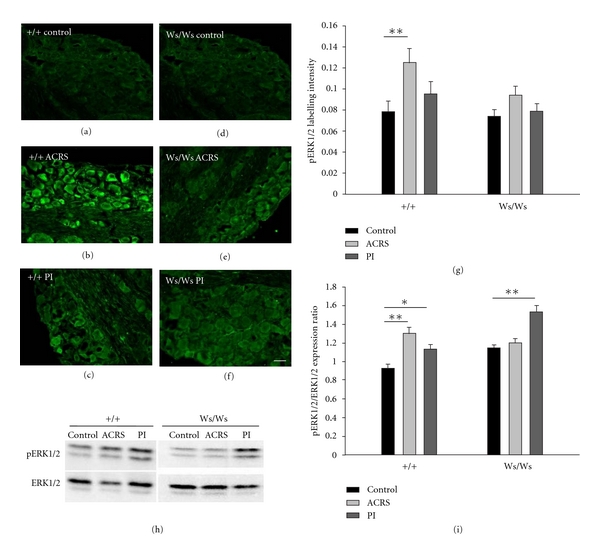
Role of ERK phosphorylation in +/+ and Ws/Ws rats induced by intestinal infection (PI) and acute cold restraint stress (ACRS). (a–f) Representative immunofluorescence images of pERK1/2 immunoreactivity- (IR-) positive neurons in L6S1 DRGs in +/+ rats (a–c) and Ws/Ws rats (d–f). Scale bar: 100 *μ*m. (g) Quantification of pERK1/2 IR labelling intensity in L6S1 DRGs. (h) Representative western blot for phosphorylated ERK1/2 in L6S1 DRG extracts using a phospho-ERK specific Ab (pERK1/2, upper panel). Protein loading was confirmed by reprobing the membrane with ERK1/2 Ab (lower panel). (i) Quantitative analysis of phosphorylated ERK1/2 protein. Data was expressed as normalized density to ERK1/2. *P < 0.05; **P < 0.01.

 In this study, we determined pERK1/2 expression in L6S1 DRGs using immunofluorescence. It was found that ACRS mediates a 0.6-fold increase in pERK1/2 immunoreactivity- (IR-) positive neurons in L6S1 DRGs in +/+ (*P* = 0.001) but not Ws/Ws rats (Figures 4(a)–4(g)). ERK phosphorylation was further confirmed by western blot with a phospho-ERK1/2 antibody. The mean immunoblot band density for pERK1/2 was greatly increased not only in +/+ rats induced by ACRS (*P* < 0.01) and *T. spiralis* intestinal infection (*P* < 0.05) but also in Ws/Ws rats induced by *T. spiralis* intestinal infection (*P* < 0.01). However, ACRS seems to be inoperative in visceral hyperalgesia in Ws/Ws rats (Figures 4(h) and 4(i)).

### 3.5. The Increased TRPV1 Expression Induced by *T. spiralis* Intestinal Infection and ACRS in L6S1 DRGs in +/+, but Not Ws/Ws, Rats

**Figure 5:**
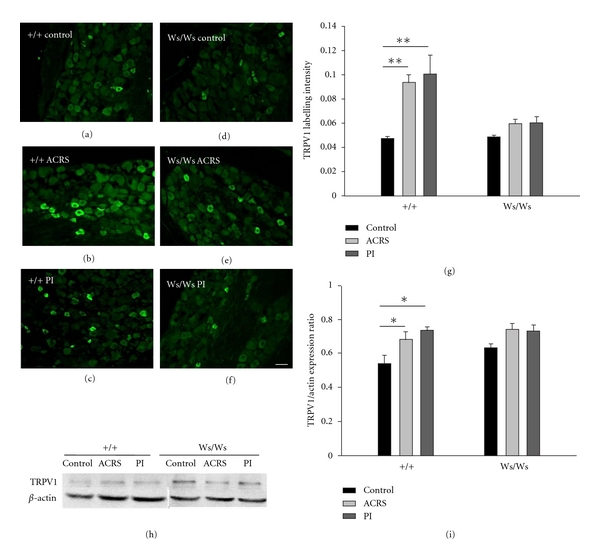
The expression of TRPV1 in L6S1 DRG neurons in +/+ and Ws/Ws rats induced by intestinal infection (PI) and acute cold restraint stress (ACRS). (a–f) Representative immunofluorescence images of TRPV1 immunoreactivity (IR)-positive neurons in L6S1 DRGs in +/+ rats (a–c) and Ws/Ws rats (d–f). Scare bar: 100 *μ*m. (g) Quantification of TRPV1 IR labelling intensity in L6S1 DRGs. (h) Representative western blotting for TRPV1 in extracts from L6S1 DRG extracts in +/+ and Ws/Ws rats. (i) Quantitative analysis of TRPV1 protein. Data was expressed as normalized density to *β*-actin. *P < 0.05; **P < 0.01.

Immunofluorescence was used to assess the expression of TRPV1 in L6S1 DRG neurons. As shown in Figures 5(a)–5(g), in +/+ rats, the cytoplasmic TRPV1 IR signal was (0.047 ± 0.002) in L6S1 DRGs from control animals, whereas it was significantly increased to 0.09 ± 0.007 (*P* = 0.004) in ACRS group and 0.100 ± 0.016 (*P* = 0.001) in PI group. No changes were observed in TRPV1 IR signal in Ws/Ws rats. The TRPV1 expression was further confirmed by western blot analysis. Compared with control group, the mean immunoblot band density for TRPV1 was also greatly increased not only in the ACRS group (*P* < 0.05) but also in the PI group (*P* < 0.05) in +/+ rats. In contrast, there is no significant difference observed in TRPV1 protein levels in Ws/Ws rats (Figures 5(h) and 5(i)).

## 4. Discussion

In this study the proposed MCs deficient rats (Ws/Ws), triggered by transient *T. spiralis* intestinal infection and ACRS, have been used to investigate the effects of MCs on the visceral hyperalgesia. In our study, we found that *T. spiralis* intestinal infection and ACRS can cause visceral hyperalgesia in wild control rats. However, its lose effect on visceral hyperalgesia induced by ACRS in Ws/Ws rats. In our previous study we have demonstrated that the number of MCs was enhanced in rats induced by infection. However, stress could not stimulate the hyperplasia of MCs. Furthermore, when we observed the MCs with transmission electron microscopy, we noticed that, in the wild control rats, the MCs of the control group and infection group retained a full complement of electron-dense secretory granules, while those of the stress groups underwent piecemeal degranulation [15]. Thus we deduced that visceral hyperalgesia induced by ACRS is dependent on MCs, although the accurate pathogenesis is still unknown.

We investigated the role of PAR2 and NGF in rats due to their close relationship with MCs. MCs are some important proinflammatory cells, which not only participate in host-defense immune responses but also regulate the functions of peripheral nerves and smooth muscles in the gastrointestinal tract [16]. Upon activation, mucosal MCs released act on PAR2 to sensitize sensory afferents in the proximity [17]. PAR2, a G-protein-coupled receptor for trypsin and MCs tryptase, has been identified in colonic myocytes, enterocytes, enteric neurons, terminals of mesenteric afferent nerves, and immune cells [18]. It has been demonstrated that activation of PAR2 on the plasma membrane of nociceptive DRG neurons innervating the mouse colon leads to sustained hyperexcitability, and ERK1/2 mediates the PAR2-induced hyperexcitability [19]. Intracolonic administration of the synthetic selective PAR2 agonist in rats increases paracellular permeability and produces visceral hyperalgesia [17]. PAR2-mediated dysfunction of colonic epithelial barrier and subsequent allodynia or hyperalgesia may play an important role in the pathogenesis of IBS [20]. Our results demonstrated that the increased expression of PAR2 was induced by both *T. spiralis* infection and ACRS in distal colon in +/+ but not Ws/Ws rats. If the results of visceral pain threshold detected by AWR score are taken into account as shown in Figure 1, we concluded the upregulation of PAR2 induced by ACRS in visceral hyperalgesia dependent on the activation of MCs. Moreover, the activation of MCs plays an important role in the upregulation of PAR2 induced by *T. spiralis* infection in visceral hyperalgesia.

NGF may be produced in part by MCs and recognized as a potent immunomodulator, behaving like a bridge between neuronal and immune cells [21]. The peripheral stress mediator norepinephrine induces visceral hypersensitivity to CRD in response to heterotypic chronic stress by increasing the expression of NGF in the colon wall [22]. NGF can further sensitize afferent nociceptors directly by binding to the high-affinity receptor tyrosine kinase A (trkA) expressed on primary afferent neurons and indirectly by triggering MCs degranulation [23]. Moreover, there are interactions among them; that is, the increased NGF production can be induced by activation of MCs, whereas NGF induces histamine release from MCs [24, 25]. In addition, anti-NGF treatments were effective in preventing the motor alterations induced by the *T. spiralis* infection, that is, inhibited increased spontaneous motor activity and reversed altered response to cholecystokinin (CCK) [26]. In this study we observed that the upregulation of NGF mRNA in intestine of +/+ rats, but not Ws/Ws rats, was induced by *T. spiralis* infection and stress. If the results of visceral pain threshold detected by AWR score are taken into account as shown in Figure 1, we concluded the upregulation of NGF induced by ACRS in visceral hyperalgesia dependent on the activation of MCs. At least we can infer that the interaction between MCs and NGF plays an important role in visceral hyperalgesia. Moreover, the activation of MCs may also be involved in the upregulation of NGF induced by *T. spiralis* infection in visceral hyperalgesia. As the NGF protein level is undermeasured with western blot method, we only detected the NGF mRNA by RT-PCR. This is the deficiency in our studies.

DRG is the primary afferent neuron in the information transmission of visceral sensation. Nociceptive processing in the visceral afferent neuronal pathways is thought to be mediated primarily via ERK and TRPV1 signal pathway. Thus, in our studies the changes of ERK and TRPV1 were investigated in rats. Visceral stimuli cause prompt phosphorylation of ERK in the spinal dorsal horn that contributes to facilitation of pain sensation and is often used as an immediate marker for excitation of spinal neurons following colonic nociception [27]. ERK1/2 is involved in the transduction of NGF neurotrophic signals by interactions with TrkA [28]. ERKs are phosphorylated in the nervous system after visceral stimulation or inflammation and play roles in central sensitization and pain hypersensitivity [29]. Our results demonstrated that phosphorylation of ERK1/2 can be induced by *T. spiralis* infections; however, the ACRS evokes phosphorylation of ERK1/2 in L6S1 DRGs dependent on MCs.

The TRPV1 pathway may also plays an important role in the process of information transfer from the peripheric receptor to central nervous system. Several studies indicated that TRPV1 plays a critical effect in visceral hyperalgesia and pain in IBS which may be associated with the increased MCs [30]. And evidences indicated that stress-induced visceral hyperalgesia in rats occurs in the absence of overt inflammation and dependents on MCs degranulation and subsequent TRPV1 activation [5]. PAR2 activation has been proven to sensitize several downstream TRPV1 channels via its G-protein-coupled receptor on sensory afferent to induce hyperalgesia [31]. If the results of visceral pain threshold detected by AWR score are taken into account as shown in Figure 1, we concluded the increased TRPV1 expression in DRGs induced by ACRS in visceral hyperalgesia dependent on the activation of MCs. Moreover, the activation of MCs also plays an important role in the increased TRPV1 expression in DRGs induced by *T. spiralis* infection in visceral hyperalgesia.

## 5. Conclusions

Our results have shown that the visceral hyperalgesia can not been triggered by stress in MCs deficient rats, although both stress and infection play an important role in visceral hyperalgesia in wild control rats. We also found that the upregulation of mediators (PAR2 and NGF) and signal proteins (pERK1/2 and TRPV1) has a close relationship with the presence of MCs. Our studies provide new evidence that the activation of PAR2 and NGF via both TRPV1 and pERK1/2 signal pathway is dependent on MCs in stress-induced visceral hyperalgesia rats. And MCs also play an important role in infection-induced visceral hyperalgesia rats.
